# Two decades long-term field trial data on fertilization, tillage, and crop rotation focusing on soil microbes

**DOI:** 10.1038/s41597-025-05314-z

**Published:** 2025-06-12

**Authors:** Marie Raab, Lars Schütz, Loreen Sommermann, Doreen Babin, Ioannis Kampouris, Davide Francioli, Rita Grosch, Günter Neumann, Annette Deubel, Joerg Geistlinger, Korinna Bade, Wilfried Rozhon

**Affiliations:** 1 Anhalt University of Applied Sciences, Department of Agriculture, Ecotrophology and Landscape Development, Bernburg (Saale), 06406 Germany; 2 Anhalt University of Applied Sciences, Department of Computer Science and Languages, Köthen (Anhalt), 06366 Germany; 3 Otto von Guericke University Magdeburg, Faculty of Computer Science, Magdeburg, 39106 Germany; 4 Julius Kühn Institute (JKI), Federal Research Centre for Cultivated Plants, Institute for Epidemiology and Pathogen Diagnostics, Braunschweig, 38104 Germany; 5 Hochschule Geisenheim University, Department of Soil Science and Plant Nutrition, Geisenheim, 65366 Germany; 6 University of Hohenheim, Institute of Crop Science (340h), Stuttgart, 70593 Germany; 7 Leibniz Institute of Vegetable and Ornamental Crops, Großbeeren, 14979 Teltow-Fläming, Germany

**Keywords:** Microbial communities, Agriculture

## Abstract

Agricultural long-term field trials provide fundamental data on crop performance and soil characteristics under diverse management practices. This information represents essential knowledge for upcoming challenges in food and nutrition security. Data provided here have been compiled since 2004 from a nitrogen(N)-fertilization intensity, tillage, and crop rotation field trial in Central Germany including standardized metrics regarding soil management, physical soil properties, crop management, crop characteristics, yield, and harvest quality parameters. In 2015, the field trial became a member of the German Agricultural Soil Research Program BonaRes. Numerous measurement results were added including plant physiology and soil and rhizosphere microbiology. DNA of bacterial/archaeal and fungal microbiomes was sequenced in the rhizosphere and root-associated soil following a meta-barcoding approach. Taxonomic and relative abundance data were included in the dataset. The dataset is the first to include information on root characteristics, soil and rhizosphere microbiomes, and crop gene expression. We encourage reuse of these biological field trial data in terms of meta-analysis, modeling and AI approaches.

## Background & Summary

In light of the increasing negative impact of land use and climate change on biodiversity, capturing processes and patterns is a crucial component in current ecological research^1^. The combination of conventional and modern monitoring methods allows the creation of extensive datasets on biotic and abiotic factors that act on various taxonomic groups over long time periods and different spatial scales^2^. To create prediction models and generate reliable forecasts about the impact of climate change on biodiversity in agroecosystems, it is important to analyze essential biodiversity variables such as species abundance and distribution, community structure, as well as ecosystem functions and services^3^. Agricultural soils are complex and dynamic ecosystems. Various microorganisms inhabit these soils, yet their role as integral components of soil^4^ remains largely unacknowledged in agricultural practice, despite their activity and functionality being of significant importance. Birkhofer *et al*.^5^ demonstrated that crop and soil management influence the structure of soil microbial communities. Several studies indicate that intensive agricultural practice negatively impacts the biodiversity of soil microbes^6,7^. Furthermore, Van Elsas *et al*.^8^ demonstrated that high microbial diversity can inhibit or delay soil colonization by plant pathogens. While the understanding of fundamental relationships has increased in recent years, comprehensive knowledge on different soil types and agricultural practices that act on soil microbiomes and their effects on crop productivity are still lacking^9–11^.

The data provided here were generated from a long-term field trial initiated in 1992 in Bernburg, Central Germany. It focuses on tillage, N-fertilization intensity, and crop rotation by comparing conservation tillage (cultivator) and conventional tillage (plough), managed either intensively or extensively, regarding N-supply and pesticide use. The trial employs a strip-split-plot design with five rotated crops including winter rapeseed, winter barley, winter wheat, grain maize, and winter wheat. The crop rotation effect can be studied in winter wheat which is grown twice with two different preceding crops (grain maize and winter rapeseed). The dataset offers comprehensive insights into microbial communities within agricultural soils. It may help to reveal intricate relationships between biotic and abiotic soil processes, and crop performance. By analyzing the dataset, researchers can gain a deeper understanding of how agricultural practice influences microbial communities in the soil and rhizosphere. Moreover, it provides valuable information for the development of sustainable agricultural strategies and integrated disease management to enhance crop productivity and mitigate environmental stress.

In 2004, the trial was restructured, and weed control variants were eliminated. The major treatments remained unaffected since the start of the trial. However, the designation of plots, and subplots never changed since 2004^12^. All experimental checkpoints were compiled since then. Agricultural field data include year, crop variety, seeding density, the timeline of sowing, emergence and harvest dates, plant protection (type and amount of pesticides and application dates), tillage dates, N-fertilization intensity and basic fertilization (macronutrients / micronutrients) amounts and dates, soil nutrient analyses in three depths, soil pH, yield, thousand kernel mass, and kernel quality (protein, starch, and oil contents). The agricultural field data (2004–2023) have been complemented by a detailed dataset generated in the frame of the German Agricultural Soil Initiative BonaRes (2015–2023). This dataset includes results on root morphology, plant nutrient status and stress metabolites (shoot and root), plant gene expression (leaf), and bacterial/archaeal and fungal communities in the rhizosphere and root-associated soil as well as root inoculation with plant beneficial microorganisms (BMs). All data are available at the BonaRes Repository for Soil and Agricultural Research Data. Daily weather data can be retrieved from the Deutscher Wetterdienst (DWD, German weather service). Since 1961, there is a meteorological station adjacent to the field. Raw DNA amplicon sequences for bacteria/archaea and fungi can be found at the NCBI Sequence Read Archive (SRA) or the EMBL European Nucleotide Archive (ENA), respectively. Data on soil porosity (computer tomography) are available at the Helmholtz Center for Environmental Research (UFZ) and metagenome data from the maize rhizosphere can be obtained at the Sequence Read Archive (SRA). For the additional data, we refer to Table 1. The BonaRes initiative is embedded in attempts to establish a world-wide network on agricultural soil properties, particularly the European Joint Program (EJP) SOIL, Global Long-term Experiment Network (GLTEN), International Long-term Ecological Research (ILTER), and International Organic Nitrogen Fertilization Experiment (IOSTV).

**Table 1:** Additional publicly available data, collected in the field trial Westerfeld, that is not part of the presented dataset.

Data category	Data source	Note
Metagenome Data	IGZ (https://www.ncbi.nlm.nih.gov/sra)	BioProject ID: PRJNA1045550
Climate Data	DWD (https://opendata.dwd.de)	Station ID: 00445
Soil Porosity Data	UFZ (https://structurelib.ufz.de/lit)	Chernozem, agricultural crop rotation, silt loam

## Methods

Given the high diversity of the data, this section has been divided into five subsections: i) field characteristics, management, and data collection; ii) soil analysis; iii) soil microbiology; iv) plant biochemistry; and v) transformation of raw data. The data collection methods are detailed in the first four subsections, while the data transformation methodology is addressed in the final subsection.

### Field characteristics, management, and data collection

The field (E 11.702, N 51.819, altitude 138,48 m; WGS84 reference system) has been managed by the working group Arable Farming of Anhalt University of Applied Sciences, Bernburg, Germany. The climate is semi-arid with a long-term average (1991–2020) of 516 mm precipitation and an average temperature of 10.1 ^°^C. The soil is a loess chernozem (pH 7.0–7.4) over limestone with an effective rooting depth of 1 m. No irrigation measures were applied, and harvest residues remained on the field. All agricultural activities were documented for each crop (so called working calendars). Additionally, handwritten field notes were kept, which facilitated cross-validation and filling of missing data while preparing the present dataset. This comprehensive data collection enabled tracking of all agricultural measures of the field trial called Westerfeld.

**Fig. 1:**
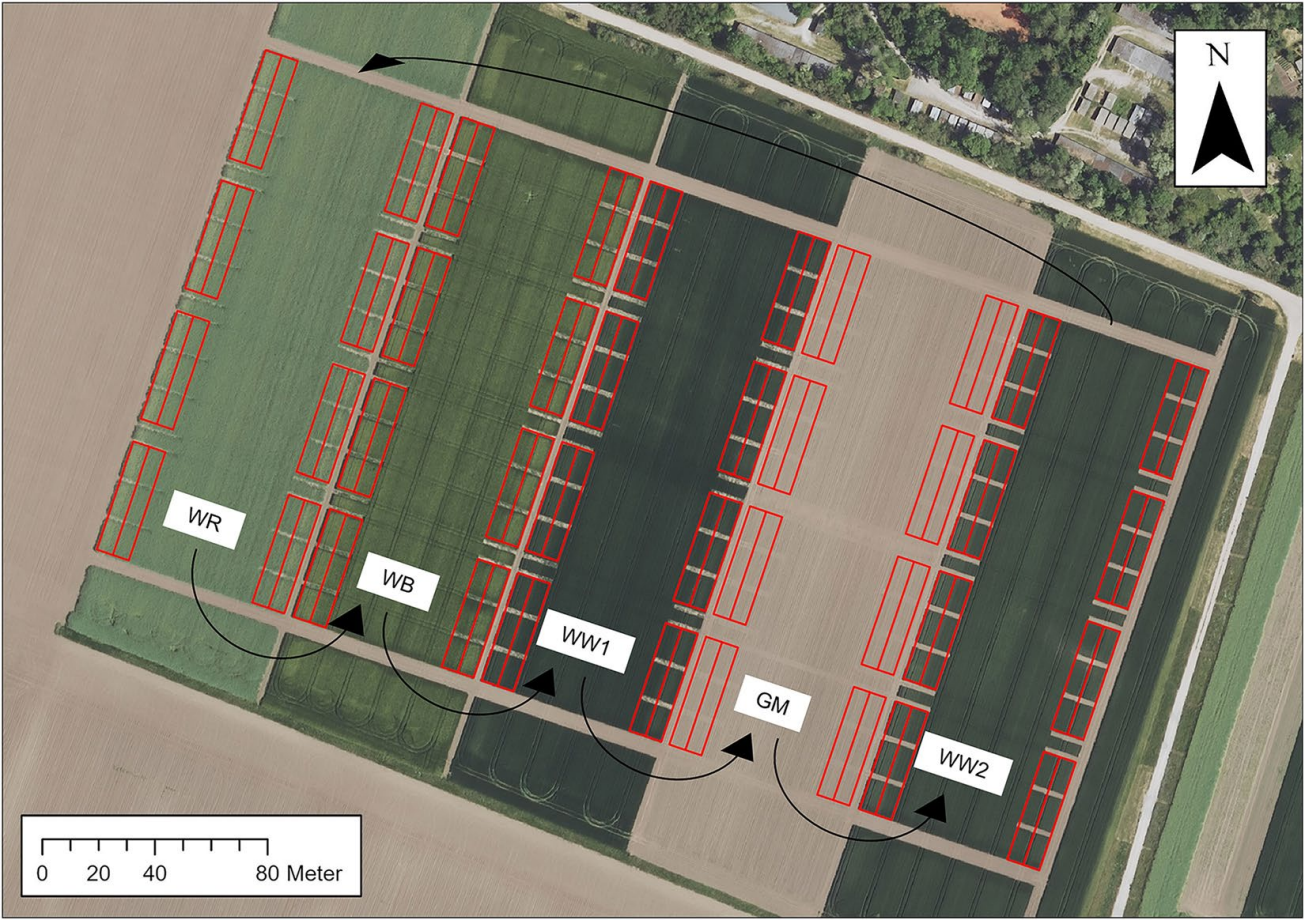
An official digital orthophoto (DOP20) outlining the field trial Westerfeld (© GeoBasis-DE / LVermGeo ST, licensed under “Data license Germany - attribution - Version 2.0” (www.govdata.de/dl-de/by-2-0)). Information on crop rotation of winter rapeseed (WR), winter barley (WB), winter wheat 1 (WW1), grain maize (GM), and winter wheat 2 (WW2) were added manually. The black arrows indicate the direction of crop rotation, while the red parcels represent the field plan.

The field trial, presented in Fig. 1, was processed in a close-to-practice manner using conventional field machinery which necessitated the conducted split-strip-plot design, because conventional machinery cannot be operated in fully randomized setups. Tillage, fertilization, pesticide application, and sowing were performed with modern land machines that allowed for precise settings concerning tillage depth, dosage of fertilizers and pesticides or seeding amounts. Three subplots per replicate (6 × 12 m each) were harvested with a Haldrup C-65 plot combine (Haldrup GmbH, Ilshofen, Germany). The device featured a working width of 2 m, an automated sample collection, sacking, and labeling system equipped with a high-pressure turbine for a pressurized air cleaning system that purges the cutting units, transportation channels, threshing cylinder, and sieves automatically after harvesting of each parcel to avoid cross-contamination. The thousand kernel mass was determined with an Haldrup GCP-30 electronic kernel counter with infrared sensor. The total yield was recorded as dt ha^−1^ (1 dt = 100 kg) adjusted to the international standards for residual moisture content of 9% for oil crops (rapeseed) and 14% for starch crops (wheat, barley and maize), to assure global comparability of yield values.

The quality of the harvested products was determined according to the respective protocols of the Association of German Agricultural Analytic and Research Institutes (VDLUFA). The protein contents of the grains were measured by elemental analysis according to VDLUFA, Volume III^13^, method 4.1.2. The starch contents were measured by polarimetry as described in protocol 7.2.1 of VDLUFA, Volume III, and the oil content of rapeseed was analyzed according to VDLUFA, Volume III, method 5.1.1.

### Soil analysis

Every soil sample was randomly taken at flowering stage with a soil corer by combining 15 soil cores (diameter 1.5 cm) per replicate block. Cores were separated according to the analyzed soil depths, 0–30, 30–60, and 60–90 cm, followed by mixing and sieving (2 mm mesh size). Soil for quantification of ammonia and nitrate was immediately frozen and kept at -20 ^°^C until analysis. Soil dry matter (%) was determined by drying fresh soil at 65 ^°^C until constant weight.

For determining the total macro- and micro-nutrient content including sodium (Na), potassium (K), phosphorus (P), magnesium (Mg), iron (Fe), manganese (Mn), copper (Cu), zinc (Zn), and boron (B), 2 g of dried and sieved soil were mixed with 21 ml of hydrochloric acid 37 % (w/w) and 7 ml of nitric acid 65 % (w/w) and refluxed for 2 h. Subsequently, the reaction was filtered and topped up with water to a final volume of 100 ml. To determine the plant available contents of Mg, Na, and micronutrients, the dried soil (10 g) was extracted with 100 ml of a mixed solution of 10 mM calcium chloride and 2 mM diethylenetriaminepentaacetic acid (DTPA) with an extraction ratio of 1+10 (m+V) (VDLUFA, Volume I, method A 6.4.1). After dilution the elements were determined by atomic absorption spectroscopy using a contrAA 700 AAS (Analytik Jena, Jena, Germany) using either graphite furnace (for B) or flame atomization (all other elements).

Plant available K, P, and Mg were extracted from 2 g dried and sieved soil with 100 ml DL buffer (19.5 mM calcium lactate, adjusted to pH 3.6). Plant available K and P were determined using the modified CAL method (Zorn and Krause (1999)^14^; VDLUFA, Volume I^15^, method A 6.2.1.1). After filtration and dilution, K and Mg were quantified by AAS (see above), while P was determined by spectrophotometry as described previously (VDLUFA, Volume I, method A 6.2.1.2). Nitrate and ammonia were analyzed by spectrophotometry according to the methods VDLUFA, Volume I, methods A 6.1.1.1 and A 6.1.2.1, respectively. Soluble sulfur (S) was determined according to VDLUFA, Volume I, method A 6.3.1 and expressed in mg sulfate (SO_4_) per 100 g dry soil.

Total nitrogen and total carbon were analyzed by elemental analysis using a CN828 analyzer (LECO, St. Joseph, MI, USA) according to method VDLUFA, Volume I, methods A 2.2.5 and A 4.1.3.1, respectively. The carbonate-C content was measured according to VDLUFA, Volume I, method A 5.3.1 and expressed as g kg^−1^ lime (calcium carbonate). The organic carbon was calculated as the difference between total carbon and carbonate carbon. The humus content was calculated from the total organic carbon content using a conversion factor of 1.724. When drying the samples, the soil water content was determined gravimetrically at the time of sampling (start of vegetation and after harvest). It was expressed as a percentage of the plant available (usable) field capacity (FC%). The total FC is 23%, the permanent wilting point (PWP) is 7%. The FC% represents the difference between FC and PWP. Data are available from different soil depths (0–15, 15–30, 30–60, and 60–90 cm).

### Soil microbiology

Soil microbial diversity was studied in two habitats. Field soil associated to roots was obtained by excavating the root systems of sample plants in each treatment and replicate at flowering stage (three plants for maize and rapeseed, 10 tillers for wheat), and shaking off the soil loosely adhering to the roots. For obtaining the rhizosphere samples, the same roots were briefly rinsed with sterilized tap water, to further remove loosely adhering soil, and then subjected three times for one minute to a Stomacher 400 Circulator (Seward Ltd, Worthing, UK) to obtain microbes from the close vicinity of fine roots^16^. Field soil (500 mg) and rhizosphere pellets were extracted with a FastPrep-24 bead beating system and FastDNA Spin Kit for Soil (MP Biomedicals, Santa Ana, CA, USA). DNAs were further purified with the GeneClean Spin Kit (MP Biomedicals). Prokaryotic 16S rRNA gene fragments were amplified using the PCR primer pair 341F (5′-CCTAYGGGRBGCASCAG-3′) and 806R (5′-GGACTACNNGGGTATCTAAT-3′) modified by Sundberg *et al*.^17^ and Caporaso *et al*.^18^, respectively, targeting the V3–V4 region of the 16S gene for both Bacteria and Archaea kingdoms. Paired-end amplicon sequencing was conducted on the Illumina MiSeq or NovaSeq 6000 platforms (2 × 250 bp; Illumina Inc., San Diego, CA, USA). Eukaryotic (Fungi) community analysis was performed by amplifying the Internal Transcribed Spacer 2 region (ITS2) using the PCR primer-pair ITS86F (5′GTGAATCATCGAATCTTTGAA-3′^19^) and ITS4 (5′-TCCTCCGCTTATTGATATGC-3′^20^). ITS2 fragments were sequenced on the Illumina MiSeq platform (paired-end mode, 2 × 300 bp).

For taxonomic classification of bacterial and fungal DNA sequences, no prior clustering was performed, but every single sequence was parsed by using the basic local alignment search tool (BLAST) algorithm^21^. The 16S reads were trimmed from adapter, barcode and primer sequences by using cutadapt^22^ (v3.7). Paired-end reads were merged using the DADA2 pipeline^23^ (v1.26.0) with R (v4.2.2)^24^. The obtained sequences were classified to the lowest possible taxonomic level by using a naive Bayes classifier^25^, trained on the SILVA SSU Reference Taxonomy database^26^ (v138.1) with ≥97% similarity and ≥80% confidence thresholds. Sequences originating from chloroplasts or mitochondria were removed. For fungal community analysis adapter, barcode and primer sequences were trimmed with the FASTX-Toolkit (https://github.com/agordon/fastx_toolkit) with paired-end sequence merging using FLASH^27^. Fungal ITS2 sequences were taxonomically assigned based on database-dependent strategy^28^ using the GALAXY bioinformatics platform and the UNITE database^29,30^ (2015–2019: v8.0, 2020–2021: v9.0) as described in Behr *et al*.^31^. The quality settings for alignment with the UNITE database were an e-value of ≤0.001, a minimal alignment length of ≥200 bp and a minimal similarity of ≥97%. Raw DNA sequences can be accessed in the sequence read archives via the respective bio-project numbers present in the dataset.

Inoculation experiments with plant-beneficial microbes were conducted with a beneficial microbial consortium (BMs) composed of two bacterial and one fungal strain (*Bacillus atrophaeus* ABi03, *Pseudomonas sp*. RU47 and *Trichoderma harzianum* OMG16) on maize plants. The strains were deposited at the German Collection for Microorganisms and Cell-cultures (DSMZ, Braunschweig, Germany) with the respective accession numbers DSMZ 32285, DSMZ 117411, and DSMZ 32722. ABiTEP GmbH (Berlin, Germany) provided a spore suspension of a rifampicin-resistant ABi03 strain, OMG16 was prepared according to Hafiz *et al*.^32^, and RU47 was grown in R2A broth (sifin diagnostics, Berlin, Germany) supplemented with rifampicin (75 μg ml^−1^; Th. Geyer GmbH, Germany) on a rotary shaker (200 rpm) for 24 h at 28 ^°^C. The concentration of each strain was adjusted with sterilized tap water immediately before drenching the maize root systems of 33 individual plants per treatment and replicate. Target plants were treated twice with 50 ml BMs suspension (10^8^ colony forming units per ml for each BM) two and five weeks after emergence. Sterilized tap water (50 ml) was used for control plants.

### Plant biochemistry

Dried shoot tissue from plants at flowering stage was ground, and 200–500 mg material subjected to micro wave-assisted digestion (Mars 6, CEM, Charlotte, USA) with 5 ml HNO_3_ (65%) and 3 ml H_2_O_2_ (30%) for 25 min at 210 ^°^C. Plants were sampled at flowering stage due to its significance as a transitional phase, where the plants shift from vegetative to generative growth. Calcium (Ca), Na, K, P, Mg, S, Fe, Mn, Cu, and Zn contents were determined via inductively coupled plasma-optical emission spectrometry (ICP-OES, Thermo Fisher Scientific, Waltham, USA). Total carbon (C) and N were determined via elemental analysis (Elementary Vario El cube, Elementar, Langenselbold, Germany). Shoot fresh matter (SFM) was obtained by separating roots from shoots with a sharp scalpel and recording the average shoot weight of three plants for maize and rapeseed or 10 tillers for wheat per treatment and replicate (g plant^−1^). Shoot dry matter (SDM) was estimated by drying the same shoots for ten days at 65 ^°^C and recording their weight a second time^33^.

The plant hormones indole acetic acid (IAA), gibberellic acid (GA_3_), cytokinin (CK, zeatin), abscisic acid (ABA), jasmonic acid (JA), and salicylic acid (SA) were analyzed from shock-frozen root material. UHPLC-MS analysis of phytohormones was carried out on a Velos LTQ System (Thermo Fisher Scientific, Waltham, MA, USA) utilizing a Synergi Polar column, 4 μm, 150 × 3.0 mm (Phenomenex, Torrance, CA, USA) as described by Moradtalab *et al*.^34^. The 1,1-diphenyl-2-picrylhydrazyl radical (DPPH) method was used to evaluate the free radical scavenging activity of antioxidants in root tissue^31,34^. Hydrogen peroxide levels were determined at 390 nm as described previously^35^. Ascorbate peroxidase (APX, EC 1.11.1.11) activity was recorded according to the method described by Boominathan and Doran^36^. Extraction and determination of superoxide dismutase (SOD, EC 1.15.1.1) was performed according to the optimized method of Moradtalab *et al*.^35^. Glycine betaine determination was performed according to the method of Grieve and Grattan^37^ with modifications described by Valadez-Bustos *et al*.^38^.

Succinic acid (chemo-attractant for rhizosphere microbes), trehalose (osmo-protectant), asparagine (chemo-attractant), proline (stress-indicator), tryptophan (auxin precursor), and total phenolics (antimicrobial) were quantified in root exudates. The samples were obtained by the installation of root observation windows in the field^31^. Soil profiles (50 × 50 cm) were cut with steel plates next to young field plants (two weeks after emergence in maize and in spring at start of the vegetative growing phase in winter wheat), covered with plastic panes and stabilized with field soil. After six weeks, roots were growing along the plastic pane. After removal of the pane, sorption filter discs (MN818, diameter 5 mm, Machery & Nagel, Düren, Germany) were placed on apical root zones (1–2 cm behind the root tips) of 3 individual roots for maize and 6 individual roots for wheat. After four hours, the filter discs were removed and transferred to 2 ml methanol (80% v/v) to extract the absorbed low molecular weight compounds. Filter discs were also placed on soil without visible root contact in order to subtract background noise. Succinic acid and trehalose in the methanol extract were analyzed according to Windisch *et al*.^39^. Determination of asparagine, tryptophan and proline was performed by HPLC-MS, using a Velos LTQ System (Thermo Fisher Scientific, Waltham, MA, USA) equipped with an ACCUTAG column, 4 mm particle size, 150mm × 3.9mm (Waters, Milford, MA, USA) after derivatisation with the ACCU-tag^40^. In brief, the methanolic extract was mixed with 15 μl of derivatization agent (ACCQFLUOR REAG, Waters, Milford, MA, USA) and 65 μl of borate buffer. After incubation at 55 ^°^C for 10 min, 400 μl acetonitrile:H_2_O (1:4) were added and the samples analyzed by HPLC-MS. Total phenolics were determined in the plant tissue according to Windisch *et al*.^39^ and total pehonlics in root exudates were determined by HPLC-MS according to Behr *et al*.^31^.

The WinRHIZO Pro root image analysis system (Regent Instruments, Quebec, Canada; software release v2009c) was used to measure total root lengths (TRL) of excavated root systems by optical scanning. Briefly, harvested roots were washed and stored in 30% (v/v) ethanol and then subsequently spread in a film of water in transparent trays, and images were taken with a flat-bed scanner (Epson Expression 10000 XL). Afterwards, the roots were oven-dried at 65 ^°^C for ten days in order to assess their dry weights. For determination of the relative arbuscular mycorrhizal fungi (AMF) colonization density, a modified staining method after Vierheilig *et al*.^41^ was used^42^. After sampling at the flowering stage, roots were cut into segments of 1–2 cm and incubated at 90 ^°^C for 45 min in 10% (w/v) KOH. Bleached roots were acidified with 2% HCl and then stained in a 5% ink-vinegar solution [5% (v/v) ink in 5% (v/v) acetic acid] at 90 ^°^C for 10 min. Destaining was performed with acidified tap water. The grid-line intersection method by Giovanetti and Mosse^43^ was employed to determine the rate of mycorrhization.

For gene expression analysis, fresh samples from the youngest fully developed leaves at flowering time (three plants per replicate) were pooled and soaked overnight in RNAlater solution (Thermo Fisher Scientific) at 4 ^°^C and then stored at −20 ^°^C. After thawing, the excess RNAlater solution was removed, and the samples were immediately submerged in liquid nitrogen and pulverized. Approximately 100 mg of this material were subjected to the RNeasy Plant Mini Kit (Qiagen GmbH, Hilden, Germany) for RNA extraction. After RNA quantification by a NanoDrop spectrophotometer (Thermo Fisher Scientific), cDNA was synthesized from 2 μg of total RNA with the High-Capacity cDNA Reverse Transcription Kit including RNase Inhibitor (Applied Biosystems, Foster City, USA). The selected marker genes were associated with biotic and abiotic plant stress responses, e. g., genes involved in signaling-pathways mediated by salicylic acid, jasmonic acid and ethylene, and the first line of defense (MAP kinases, ascorbate peroxidase, superoxide dismutase). In addition, several plant genes involved in N-metabolism and Fe-transport were analyzed. The reference genes coding for ubiquitin (Ubi) and elongation factor 1 *α* (EF1 *α*) were used for normalization in wheat (Table S1) and Ubi and actin (ACT) were used in maize (Table S2). The target and endogenous control reference genes were validated and only primers with 100  ± 10% efficiency were used. The Power SYBR Green Supermix (Thermo Fisher Scientific) for qPCR and a peqSTAR 96Q thermal cycler (PEQLAB Biotechnologie GmbH, Erlangen, Germany) were applied for three technical replicates as described previously^44^. The Δ*C*_T_ method was applied to determine relative transcript abundances. The averaged data of the three technical replicates were first normalized to the averages of the endogenous controls and then logarithmically transformed to fold change differences (${\log }_{2}{\rm{FC}}$). The ΔΔ*C*_T_ values^45^ can be obtained by the allocation of different treatments, e. g., BM inoculation vs. control.

### Transformation of raw data

Data collection methods were applied by several people at different times, resulting in highly heterogeneous raw data within this dataset. At the beginning of the field trial, there was no specific documentation requirement, leading to the lack of a standardized data format. Consequently, data were independently documented in various formats, including handwritten calendars, Microsoft Word documents, and Microsoft Excel spreadsheets with different designs. Following a thorough review of all available data, a unified data model was developed to cover all existing data. This process involved creating multiple data tables and reorganizing pieces of information by separating distinct data elements into individual columns within the data tables. The original data were transformed into this new model. The Microsoft Word documents and handwritten paper documents were manually processed, while existing data tables in other formats were converted using custom Python scripts. Given the diversity of formats resulting from the contributions of different individuals, multiple Python scripts were developed to harmonize the data and align it with the target structure.

Additional columns were added to improve the clarity and readability of the data. On the one hand, data categories were introduced to assign plant genes to a gene expression category and plant protection measures to a plant protection product type. On the other hand, the quantities were distributed across several columns for better organization. For example, a distinction was made between liquid and solid products for plant protection, while fertilization data were divided into nitrogen (N), phosphorus (P), and potassium (K).

We also cleaned the taxonomic data of the microbial communities by addressing entries labeled as unknown, unidentified, or unclassified. For such entries, we identified the highest reliable taxonomic rank and populated the subsequent columns by appending “_sp” for species, “_gen” for genus, “_fam” for family, “_ord” for order, “_cls” for class, and “_phy” for phylum, ensuring clear identification of incomplete taxonomic rankings. Additionally, missing data were supplemented with information from the UNITE and SILVA databases, bringing the data table up to date with the latest advancements in microbial diversity research.

In some data records, the distinction between the three sub-replications per replicate block was not recorded. Consequently, a single value per block and treatment was provided. In these cases, the same value was assigned to each sub-replication. As a result, the mean value per block corresponds to the value of the raw data. More specifically, the Plot_IDs 1–80, 81–160, and 161–240 were assigned identical values.

## Data Records

The dataset is available at the BonaRes Repository^46^, with each data table available for download in CSV format. Comprehensive metadata have also been published in the BonaRes Repository. The website provides a detailed summary of each data table, including descriptions of columns, units, and data types, which can optionally be converted to and downloaded as a PDF file. In the following, we will show the main aspects of the data description. For further details, readers are encouraged to consult the BonaRes Repository.

Each data record contains the experimental results of a long-term agricultural field trial on a yearly basis. The whole dataset was organized into data tables. They can be mapped to the following different semantic data categories: i) experimental data; ii) field data; iii) microbial communities; iv) soil data; and v) plant data. The subsequent subsections focus more specifically on the individual data categories. For a complete overview of all data tables, including the number of features and the number of observations of each data table, we refer to Table 2.

**Table 2:** Numbers of observations and features per data table of the dataset (* includes the Archaea data).

Data category	Data table	#Observations	#Features
Experimental Data	CROP	6	5
Experimental Data	CROP_ROTATION	101	5
Experimental Data	EXPERIMENTAL_SETUP	4800	7
Experimental Data	FACTOR	2	3
Experimental Data	FACTOR_1_LEVEL	2	5
Experimental Data	FACTOR_2_LEVEL	2	5
Experimental Data	PLANT_VARIETY	38	4
Experimental Data	PLOT	240	8
Experimental Data	REMARK	9	3
Experimental Data	SEED_STOCK	38	2
Experimental Data	TREATMENT	4	3
Field Data	FERTILIZATION	12527	8
Field Data	FERTILIZER	7	3
Field Data	HARVEST	4716	5
Field Data	PLANT_PROTECTION	27536	8
Field Data	PLANT_PROTECTION_PRODUCT	134	4
Field Data	PLANT_PROTECTION_PRODUCT_TYPE	7	3
Field Data	SOWING	4728	7
Field Data	TILLAGE	4728	6
Field Data	TILLAGE_MEASURE	2	3
Field Data	YIELD	4716	8
Microbial Communities	FUNGI	299966	18
Microbial Communities	BACTERIA*	771361	18
Microbial Communities	BIOPROJECT	10	2
Microbial Communities	HABITAT	2	3
Microbial Communities	BENEFICIAL	2	3
Microbial Communities	KINGDOM	3	2
Microbial Communities	PHYLUM	91	3
Microbial Communities	CLASS	264	3
Microbial Communities	ORDER	629	3
Microbial Communities	FAMILY	1218	3
Microbial Communities	GENUS	3025	3
Microbial Communities	SPECIES	6564	3
Soil Data	SOIL_LAB	3866	25
Soil Data	SOIL_SAMPLING	3866	7
Plant Data	GENE_EXPRESSION	3520	8
Plant Data	GENE_EXPRESSION_CATEGORY	5	3
Plant Data	PLANT_LAB	224	17
Plant Data	PLANT_SAMPLING	224	5
Plant Data	ROOT	112	25

**Fig. 2:**
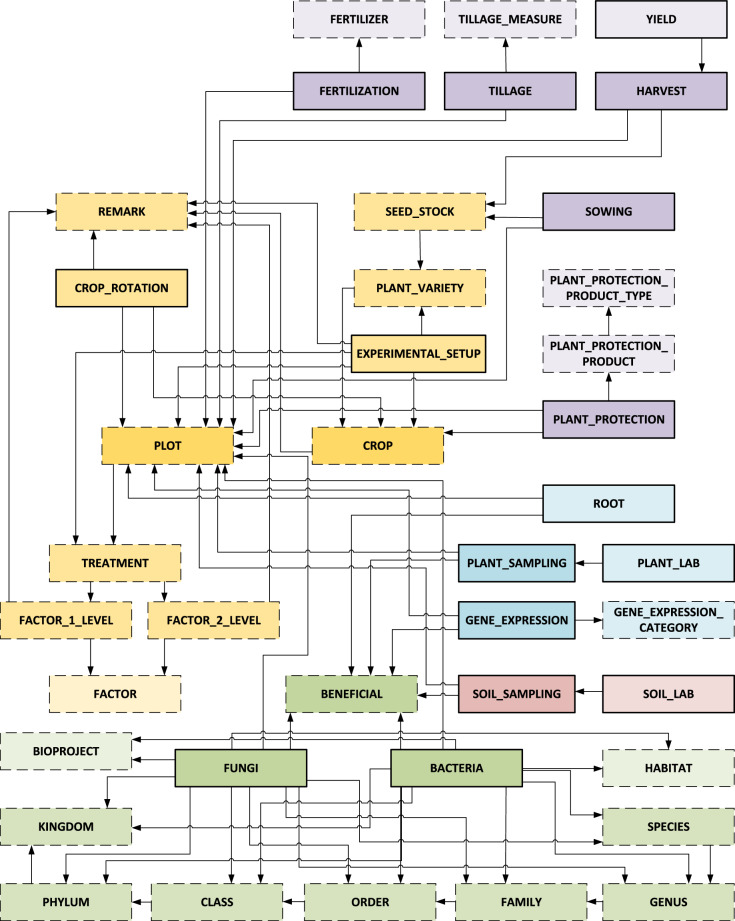
Data tables and their relationships. Each colored box represents a data table, and the arrows indicate their relationships. The different data categories are visually encoded by color (experimental data (yellow), field data (purple), microbial communities (green), soil data (red), and plant data (blue)). The color grading depends on the number of relationships, and the frame style represents the type of data table (data tables with attributes only (dashed line), data tables with experimental results (solid line)).

The prefix “lte_westerfeld.V1_0_” has been applied to all data tables, and their names were set in uppercase. Figure 2 depicts how the data tables relate to each other. While each box represents a data table, the connections between the data tables are visualized by arrows. The direction of the arrows indicates to which data table is being referred to. Each outgoing arrow from a data table represents a foreign key that relates to another data table. This means that data records from one data table can be linked to data records from other data tables. This essentially results in two different types of data tables. First, there are data tables that store the different, unique attributes (e. g., FERTILIZER and FAMILY). Second, there are more complex data tables that actually store the experimental results. As can be seen, each semantic data category consists of several data tables, and there are numerous connections between data tables within the data categories and also between them. The central data table is the plot data table. All experimental data are attached to it.

**Fig. 3:**
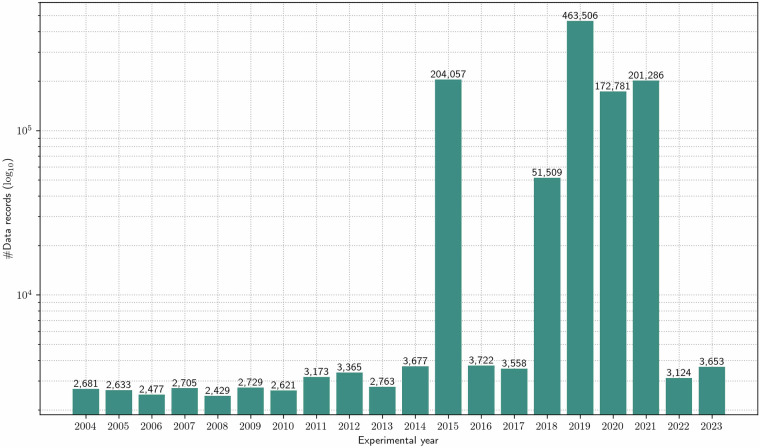
Number of data records per experimental year. This bar chart shows the data records distribution from 2004–2023.

The dataset includes varying numbers of data records for each experimental year, reflecting inconsistencies in data collection throughout the field trial’s duration. This variation arises because the data were generated by different projects, each with distinct timelines and objectives. While the field trial has been ongoing since 1992, sampling for the BonaRes project DiControl began in 2015 and concluded in 2023. As a result, data on detailed soil nutrient contents, soil microbiology, and plant biochemistry are only available from 2015 onward. An overview of the number of data records available per experimental year is shown in Fig. 3.

### Experimental Data

**Fig. 4:**
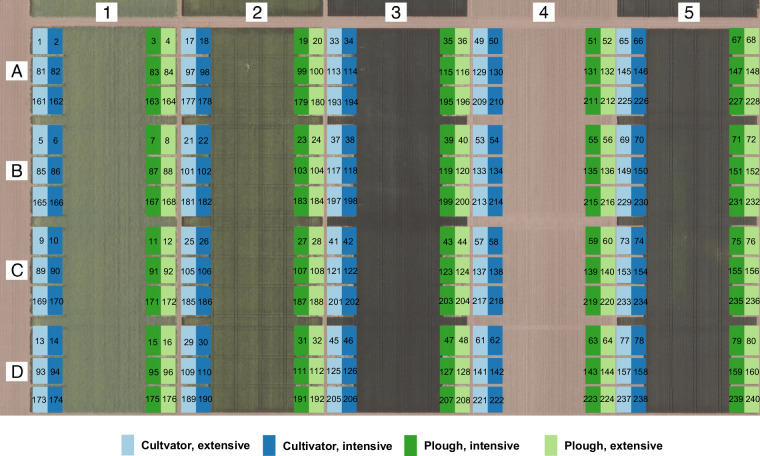
Experimental setup of the field trial Westerfeld based on an official digital orthophoto (© GeoBasis-DE / LVermGeo ST, licensed under “Data license Germany - attribution - Version 2.0” (www.govdata.de/dl-de/by-2-0)). It illustrates the arrangement of all 240 plots and highlights the four different treatments applied in the experiment. The rows are labeled with numbers 1–5, and the subplot replicates are designated by letters A–D. The numbers display the distribution of all Plot IDs across the field.

The experimental data are organized into eleven distinct data tables, which form the foundation of the entire dataset. The experimental data are about the experimental setup of the field trial (Fig. 4). The field was divided into five rows (row 1 to 5), with one specific crop planted per row and year. The crop sequence begins with winter rapeseed and continues with winter barley, winter wheat 1, grain maize, and winter wheat 2, following the crop rotation schedule at the field site. Annually, the crops shift to the next row. The parallel growth of all five crops enables the examination of each crop in every year. The crop rotation scheme is presented in Fig. 1.

Within each row, four sub-rows can be distinguished which actually represent four different treatments. These sub-rows vary according to the factors of tillage and fertilization. While the factor level 1 is divided into two tillage measures termed cultivator and plough, the factor level 2 differentiates between intensive or extensive N-fertilization. The rows are divided into four blocks (replicates A to D). Additionally, there are three sub-replications in each replicate block, resulting in a total of 240 plots (5 rows, 4 treatment sub-rows, 4 replicate blocks, and 3 sub-replications per replicate block).

The dataset also reflects the advances in plant breeding, and current cultivars were replaced by modern plant varieties every four to five years.

### Field Data

The field data pertains to agricultural activities that have been described in the subsection “Field characteristics, management, and data collection” within the Section “Methods”. This data category comprises ten data tables. These are organized into five subcategories: i) sowing; ii) tillage; iii) plant protection; iv) fertilization; and v) harvesting. Each data table includes information on the experimental year, plot ID, and the date of a corresponding treatment.

The field data tables comprehensively document all field management activities carried out throughout the year. Following the sowing of grains, the seeding rate (grains m^−2^) and plant emergence dates were documented. After tillage, data on soil depth (cm) and the corresponding tillage measure were recorded. Fertilizer applications were meticulously categorized into nitrogen (kg N ha^−1^), phosphate (kg P ha^−1^), and potassium (kg K ha^−1^). While K and P were applied as basic fertilization approximately every two years, N was supplied annually in varying amounts depending on the intensive or extensive variants. The applied plant protection products were classified into liquid (l ha^−1^) and solid (kg ha^−1^) forms and categorized as herbicides, fungicides, insecticides, or growth regulators. During harvest, yield (dt ha^−1^) and thousand kernel mass (g) were recorded. Additionally, depending on the crop, the crude protein or oil contents (rapeseed) was measured, while for maize, the starch content was additionally determined.

Two important considerations must be taken into account when using the field data for further analysis. First, from 2008 to 2010, winter rye was planted in blocks C and D, which were designated for winter barley. As a result, no data are available for winter barley in these blocks during this period, as the published dataset includes only winter rapeseed, winter barley, winter wheat, and grain maize. Second, in 2017, summer rapeseed was cultivated in the plough tillage sub-rows, while winter rapeseed was used for the cultivator tillage. This decision was made because significantly fewer winter rapeseed plants emerged in the plough variants than anticipated. To facilitate the analysis of the pre-crop effect, it was decided to abandon the sparse winter rapeseed vegetation and replace it with summer rapeseed. This substitution allowed the soil to retain essential nutrients from the rapeseed debris after harvest.

### Microbial Communities

The microbial communities data category consists of twelve data tables that contains all details related to the microorganisms bacteria/archaea and fungi. The related methods for the data collection are described in the subsection “Soil microbiology” of the Section “Methods”. Table 3 provides an overview of the available microbial community data by crop and experimental year, as such data are not available for the entire duration of the field trial.

**Table 3:** Overview of the available microbial communities per crop and experimental year (bacteria/archaea data (B), fungi data (F)).

Experimental year	Winter rapeseed	Winter barley	Winter wheat 1	Grain maize	Winter wheat 2
2015			B, F		B, F
2018			F		F
2019	B, F		B, F	B, F	B, F
2020				B, F	
2021				B, F	

The microbial communities data tables document which microbial communities were found in different soil samples and years. The results are divided into the two main tables relating to the detected fungi and bacteria/archaea respectively. In addition to crop and experimental years, the microbial communities can also be differentiated by habitat (Field_Soil and Rhizosphere) and beneficial (Control and BMs). Depending on the results of the taxonomic analysis, the characterization of the different microbes is available on different taxonomic levels ranging from kingdom to phylum, class, order, family, genus, and species. The column “Value” indicates how frequently the specific DNA sequence was found. No DNA sequences are available at the BonaRes Repository, but must be obtained from the respective sequence read archives. The BioProject ID, the Sequence ID or the related SRP/ERP numbers can be used for the retrieval of the original raw DNA sequences and related metadata. The datasets can be accessed alternatively via the SRA^47–51^ or the ENA^52–56^ databases. For fungi, it is also possible to retrieve information on the respective species through the accession number and SH (species hypothesis) code of the fungal UNITE database (https://unite.ut.ee).

Another important consideration is that different maize varieties were cultivated in blocks A, B and C, D. As a result, soil samples for the subsequent microbial analysis were only taken from blocks A and B to avoid a hidden cultivar effect. This means that the four replicates used to study the microbial communities in the maize-related data were only sampled in blocks A and B.

### Soil Data

This data category is the smallest and consists of two data tables. The related methods are described in the subsection “Soil analysis” within the Section “Methods”. These data tables describe the soil conditions of the field experiment. From 2012 to 2019, the water holding capacity (WHC), representing the plant available (usable) field capacity (FC% (v/v)), was measured at four depths (0–15, 15–30, 30–60 and 60–90 cm). Between 2019 to 2021, different physicochemical soil properties, such as zinc, iron, copper, magnesium concentrations, and others, were analyzed to assess soil fertility. During this period, additional measurements included soil dry matter (SDM), pH value, and soil organic matter (SOM), recorded at three depths (0–30, 30–60, and 60–90 cm).

It is important to note that the lime and humus content, as well as the total organic carbon content of the soil, were only measured once in 2009. Nevertheless, the data were still included in the dataset to provide an opportunity for parameterizing models that integrate soil functions, such as BODIUM^57^.

### Plant Data

The plant data category comprises five data tables, with the methods for data collection detailed in the subsection “Plant biochemistry” of the Section “Methods”. These data tables are organized into three subcategories: gene expression, roots, and the laboratory analyses of plant samples (plant laboratory data). This type of data was not collected for the entire duration of the field trial. Table 4 provides an overview of the available plant data by crop and experimental year.

**Table 4:** Overview of the available plant data per crop and experimental year (plant laboratory data (P), gene expression data (G), root data (R)).

Experimental year	Winter rapeseed	Winter barley	Winter wheat 1	Grain maize	Winter wheat 2
2019	P		P, G, R	P	P, G, R
2020				P, G, R	
2021				P	

The genes were categorized into different groups, such as stress-related genes, genes for nitrogen, phosphorus, or iron/zinc uptake, and nitrogen metabolism and transport. The column Value of gene expression displays the relative transcript abundances ($\Delta {C}_{{\rm{T}}}{\log }_{2}{\rm{FC}}$). This column is the only column that is already normalized, not presented in raw data format.

In the root dataset, various low molecular weight compounds, enzymes, and phytohormones were recorded, along with information on root length and dry weight. For plant laboratory data, various physiological plant parameters were analyzed, including shoot concentrations of zinc, calcium, copper, iron, and other elements. This data table also includes the option to differentiate between beneficial microbes (Control and BMs), that were used to inoculate maize root systems with potential effects on plant physiology.

## Technical Validation

The data were structured according to a predefined data schema required by the BonaRes Repository. We developed a data schema for Soil Data, Plant Data, and Microbe Communities, as no predefined schema existed, since this was the first time these types of data were being published at the BonaRes Repository. This process included a database normalization step for data storage purposes. This requirement of the BonaRes Repository offers several advantages, such as eliminating data redundancy, ensuring data consistency, and reducing data storage costs. However, this step requires additional quality control measures to verify the results of the processing steps. To address this, we employed a custom Python script to automatically test the correctness of the processed data. It compares the data before and after processing by checking the numbers of rows and columns, as well as the specific data values. In this context, the data were considered equivalent if their shape, i. e., the numbers of rows and columns, and values matched. The Python script generates a file highlighting any identified differences. For example, it reports differences caused by rounding errors. We ran the script and corrected the data until no differences remained. We published the custom Python script in the accompanying source code repository (see Section “Code Availability").

Furthermore, we utilized the DQ-Kit (v1.0)^58^, provided by the BonaRes Center, for data validation purposes. DQ-Kit is a Web service that checks datasets for quality issues. It also provides statistical summaries and visualizations of the data. First, we focused on the aspect of data redundancy. In the first run, only one out of nine reports raised some issues. Specifically, the DQ-Kit detected 99 duplicated observations in the data table related to the plant protection measures. These duplicates have subsequently been removed. In the final DQ-Kit run, no further issues were reported. Second, two persons who were responsible for the preparation of the final dataset independently reviewed the visualizations and statistical summaries generated by DQ-Kit. Neither found any abnormalities. All reports generated by the DQ-Kit are available in the accompanying source code repository. However, the DQ-Kit was unable to process the fungi and bacteria/archaea data due to its large size. Therefore, we checked this particular data for redundant data observations with another Python script. It found 63,377 putative duplicates in the bacteria/archaea data. Upon closer inspection, we determined that these were genuine observations with slight differences in DNA sequences not represented in the repository dataset. These sequence variations did not affect taxonomic classification or similarity values, which led to their identification as duplicates. We resolved this issue by adding operational taxonomic unit identifiers (OTU IDs) for each unique DNA sequence using a separate column in the related data table. The Python script used for this process is also included in the accompanying source code repository.

It is important to note that we did not remove presumable outlier values, as our objective was to publish the data in its most unprocessed form possible.

## Usage Notes

Each data table in the dataset includes a unique identifier column (ID column) that serves as a primary key, similar to those used in database systems. For example, the FERTILIZER data table contains the ID column Fertilizer_ID. Additionally, many other data tables include columns functioning as foreign keys, linking them to other data tables within the dataset. For instance, the FERTILIZATION data table includes a Fertilizer_ID column, allowing relationships to be established between different data tables. In this case, to obtain comprehensive fertilization information from the field trial, the FERTILIZATION and FERTILIZER data tables can be joined using the Fertilizer_ID column which serves as a primary key in FERTILIZER and a foreign key in FERTILIZATION.

To maintain clarity and ensure uniqueness, the names of the data tables and their corresponding ID columns were kept consistent. Specifically, each ID column was named after its associated data table, with a unique suffix appended. For example, the BACTERIA data table contains the ID column named Bacteria_ID. This naming convention was deliberately designed to prevent conflicts and facilitate direct association between each ID column and its respective data table. As a result, this approach improved the comprehensibility of the dataset structure.

While the transfer to a data model with many separate data tables and foreign key relationships is advantageous for data storage, these separate data tables must be merged in order to analyze individual facts. For specific analysis questions, we developed and provide several custom Python scripts that perform the required joining of certain data tables. These scripts are included in the accompanying source code repository. An overview of these scripts and the mandatory input data tables that are needed for their use is presented in Table 5. It is important to note that when adding the crop names to the data tables, a distinction must be made between winter wheat 1 and winter wheat 2. To accomplish this, the preceding crop for each winter wheat must be identified. This information is stored in the REMARK data table.

**Table 5:** Overview of available Python scripts for data preparation, along with the required input data tables for their functionality. The output is a merged data table with all relevant experimental information.

Script name	Input data tables
bacteria.py	BACTERIA, BENEFICIAL, BIOPROJECT, HABITAT
fertilization.py	FERTILIZER, FERTILIZATION
fungi.py	BENEFICIAL, BIOPROJECT, FUNGI, HABITAT
gene_expression.py	GENE_EXPRESSION, GENE_EXPRESSION_CATEGORY
harvest.py	HARVEST, YIELD
plant_lab.py	BENEFICIAL, PLANT_LAB, PLANT_SAMPLING
plant_protection.py	PLANT_PROTECTION, PLANT_PROTECTION_PRODUCT, PLANT_PROTECTION_PRODUCT_TYPE
root.py	BENEFICIAL, ROOT
soil_lab.py	BENEFICIAL, SOIL_LAB, SOIL_SAMPLING
sowing.py	PLANT_VARIETY, SEED_STOCK, SOWING
tillage.py	TILLAGE, TILLAGE_MEASURE
common.py	CROP, EXPERIMENTAL_SETUP, FACTOR_1_LEVEL, FACTOR_2_LEVEL, PLOT TREATMENT, KINGDOM, PHYLUM, CLASS, ORDER, FAMILY, GENUS, SPECIES

As one specific example, to analyze the trial involving crop rotation, fertilization, and tillage, it is recommended to join the crop data with information on Factor_1_Level (Tillage) and Factor_2_Level (Fertilization) into a single data table. This integration can be efficiently achieved using the EXPERIMENTAL_SETUP data table that documents the annual experimental setup for each plot. The important columns for this integration are Plot_ID and Experimental_Year, enabling direct access to the Crop_ID and Treatment_ID. The TREATMENT data table contains detailed information on treatments based on the combinations of the factor levels. By utilizing the Factor_1_Level_ID and Factor_2_Level_ID, the treatments from the long-term field trial can be accurately incorporated into the dataset. This process is facilitated by the function *prepare_table_experiment* within the file *common.py*. This function is invoked by the other Python scripts listed in Table 5. Additionally, these scripts replace the foreign keys with their corresponding data records, ensuring that the final data table is ready for analysis.

Since each taxonomic level of fungi and bacteria/archaea is stored in a separate data table, it is advisable to create a single comprehensive data table for the analysis of microbial communities. This data table should include the complete taxonomic string from kingdom to species, along with information on crop, fertilization, and tillage. To facilitate this process, we provide a Python script specifically designed to prepare the fungal and bacterial (including archaeal) data.

## Supplementary information

Supplementary Information

## Data Availability

No custom source code was developed to generate the dataset described in this manuscript. However, custom Python scripts were employed to transform the raw data into our single data model as described in subsection “Transformation of raw data” of the Section “Methods”. Furthermore, we used several custom Python scripts for technical validation. We published these source code files (v1.3)^59^ in a source code repository hosted on GitHub. It is important to note that these Python scripts are provided solely for documentation purposes, ensuring traceability and transparency. It is not possible to run these Python scripts because we cannot supply the raw data for them. Additionally, the source code repository also contains comprehensive usage instructions, Python scripts that represent basic usage examples, and Python scripts for preparing the comprehensive data tables as mentioned before. These two kinds of Python scripts can be applied directly to the published dataset. The DQ-Kit is a third-party Web service that uses a custom source code to validate each data table of the dataset. However, the underlying source code is not accessible. We published the DQ-Kit reports in the accompanying source code repository for documentation purposes.
