# Supplementary material for: Two decades long-term field trial data on fertilization, tillage, and crop rotation focusing on soil microbes
Source: Sci Data. 2025 Jun 12;12:986. doi: 10.1038/s41597-025-05314-z (PMC12162863; doi:10.1038/s41597-025-05314-z)
Supplement: Supplementary file 1 — Supplementary Information [file 41597_2025_5314_MOESM1_ESM.pdf]

Supplementary Information

qPCR primer lists for reference and target genes for gene expression analysis in the crops winter wheat and grain maize.

Table of Contents

**Title**	**Pages**
Table S1	2
Table S2	3 – 4

Table S1: Primer list for gene expression analysis in the crop winter wheat.

Gene<br>*Code*	Annotation	Accession<br>Reference	Forward and Reverse Primers (5'-3')
**Ubiquitin**<br>*TaUBIQ*	Reference gene	Cruz et al. 2015	CCTTCACTTGGTTCTCCGTCT<br>AACGACCAGGACGACAGACACA
**Elongation factor 1α**<br>*TaEF1α*	Reference gene	Cruz et al. 2015	ATGATTCCCACCAAGCCCAT<br>ACACCAACAGCCACAGTTTGC
β-1,3-glucanase<br>*TaPR1*	Stress-related	HQ848391	CAATAACCTCGGCGTCTTCATCAC<br>TTATTTACTCGCTCGGTCCCTCTG
Lipoxygenase<br>*TaLOX*	Stress-related	Cruz et al. 2015	CGACCCGCAGCTGTTGA<br>CCCTTGTGATCGGAGGTGTT
Allene Oxide Synthase<br>*TaAOS*	Stress-related	Wang et al. 2017	ACCGTGTTCAACAGCTACGG<br>AGCGCCTCTATCGTCACCTT
Superoxide Dismutase<br>*TaSOD*	Stress-related	JQ613154.1	CATTGTCGATAGCCAGATTCCTTT<br>AGTCTTCCACCAGCATTTCCAGTA
Catalase<br>*TaCAT*	Stress-related	GU984379.1	TTTGATGGGAGTCTTGTGCTTGTG<br>ACGGTGAGGGAGTTGTCGTTGTT
Peroxidase<br>*TaPer*	Stress-related	X53675.1	CAGCCCTGTAGCCAACATAAA<br>GCACTTCCACGACTGCTTTG
Glutathione-S-Transferase 4<br>*TaGSTU*4	Stress-related	Behr et al. 2024	TTCAAGCATCCAACCTCTCC<br>GCTGTCACATCCATCCAAAA
Glutathione-S-Transferase<br>*TaGSTZ*	Stress-related	Behr et al. 2024	CCAAGCCCATTTGTTACCAG<br>GTGGATGAGCACGGGTATCT
MYB transcription factor<br>*TaPIMP2*	Stress-related	Wei et al. 2017b	GCATTGTACGGCCAGTTCG<br>CGAGGAGGCTCTGTTCTTGG
MYB transcription factor 80 *TaMYB80*	Stress-related	Zhao et al. 2017	CAGATGCTCCTCCCTTGG<br>GTGATCCTGGTGTAGTTGC
MYB transcription factor<br>*TaODORANT1*	Stress-related	Wei et al. 2017a	CCGAAGCCCATGTACCTCC<br>CGGATCTATGATCGGTCTATGTG
WRKY transcription factor 49<br>*TaWRKY49*	Stress-related	Wang et al. 2017	CTTCCCTGCCGCATTCT<br>ACGCTCTCGCCCTAGTG
WRKY transcription factor 62<br>*TaWRKY62*	Stress-related	Wang et al. 2017	TCGTTGACCACCACCAG<br>AGCCGTCCCCAAATCCA
Nitrate Reductase<br>*TaNR1*	N metabolism	Buchner and Hawkesford 2014	GGCCAATTCYTTCATCTCCTTCTG<br>TACRTSCACAGATTGATGCGTCSA
Nitrite Reductase<br>*TaNIR*	N metabolism	Buchner and Hawkesford 2014	ACGAGGAGTAGGCCGGCTASGAG<br>ATCAGCCGCAGCCCATCTCTRC
Nitrate/Peptide Transporter Family 7.1<br>*TaNPF7.1*	N metabolism	Buchner and Hawkesford 2014	CTACAAGACCTGCGCCATCTTC<br>GATGAGGTATAGCCGCGAGGAG
Glutamate dehydrogenase 2 *TaGDH2*	N metabolism	Buchner and Hawkesford 2014	AGGATGGGAGCATTCACCTTGG<br>GGATATAAGAACTKTCATCCACCACG
Iron transporter<br>*TaVIT2*	Fe uptake	Connorton et al. 2017	CTCCCCCTACATGTTCGT<br>CCCTTGACGTAGCCGAA
Mitogen-activated Protein Kinase 3<br>*TaMPK3*	Stress-related	Goyal et al. 2018	GGAGATCAAGCTCCTCAGGC<br>ACTGGCAGTGTTCTTCCGAG
Mitogen-activated PK4<br>*TaMPK4*	Stress-related	Goyal et al. 2018	TCGAGCCTGGGATTTCTTCG<br>GTCAACAGTGATGCGTCTGC
Mitogen-activated PK6<br>*TaMPK6*	Stress-related	Goyal et al. 2018	CAGCTTATCTCCGAGGAAAACG<br>TTGTGCCGCACTAGTTGGA
Mitogen-activated Protein Kinase Kinase 6<br>*TaMKK6*	Stress-related	Goyal et al. 2018	CAGCTTATCTCCGAGGAAAACG<br>TTGTGCCGCACTAGTTGGA
Mitogen-activated Protein Kinase Kinase 10<br>*TaMKK10-1/3a*	Stress-related	Goyal et al. 2018	CTCAAGGTCCAGCACTACGG<br>CCACCAGCTCGAGGAGTAGA
Mitogen-activated Protein Kinase Kinase 10<br>*TaMKK10-1/3b*	Stress-related	Goyal et al. 2018	CCAGGGCCCTATGATCCGTA<br>TTGCCGAAGAAGATAGCGCA

Table S2: Primer list for gene expression analysis in the crop grain maize.

Gene<br>*Code*	Annotation	Accession Reference	Forward and Reverse Primers (5'-3')
**Ubiquitin-conjugating enzyme**<br>*ZmUbi*	Reference gene	NM_001154750.1	CAGGTGGGGTATTCTTGGTG<br>ATGTTCGGGTGGAAAACCTT
**Actin**<br>*ZmACT*	Reference gene	NM_001165684.1	GGAGCTCGAGAATGCCAAGAGCAG<br>GACCTCAGGGCATCTGAACCTCTC
Cytosolic ascorbate peroxidase<br>*ZmAPX1*	Stress-related	GRMZM2G316256	GCTCTGTCTTGCATGGCACTCC<br>GATGGGCTCTAGCAACCTGACG
Mitogen-activated protein kinase<br>*ZmMPK3*	Stress-related	GRMZM2G017792	ACAGCGACATGATGACGGAGTA<br>CCAATCACCTCGGTTATGAG
Nicotianamine synthase<br>*ZmNAS3*	Fe/Zn uptake	XM_008666956.4	GGCTCACCAGAAGATGGAGGAG<br>TCACGCATGTGGTGTAGACACG
Nicotianamine synthase<br>*ZmNAS4*	Fe/Zn uptake	Zhou et al. 2013	CACGGCACACACCACAAGCAACAAG<br>ATCCATGCGGTGTGGGCACATAGAC
Ammonium Transporter<br>*ZmAMT1*	N uptake	Gu et al. 2013	CCAGCAGCCAGGTGTAAAA<br>CGACTCCCAAGTAGCCAAG
Zinc transporter<br>*ZmIRTa*	Fe/Zn uptake	NM_001158638.2	CTGCAGAGCAGCGTCAGG<br>AGTACTGTGTCATGTCGTCTC
Oligopeptide transporter<br>*ZmOPT8a*	Fe/Zn uptake	GRMZM2G086258	GCTACATGAGCATGTCGCAGGCT<br>TGCCAGCCACAATGGTACCAACAAACTGA
Oligopeptide transporter<br>*ZmOPT8b*	Fe/Zn uptake	Kobae et al. 2014	GGCTACATGAGCATGGCACAGG<br>CCAGCCACAATGGTACCGACAAACTGG
Oxo-phytodienoate reductase<br>*ZmOPR8*	Stress-related	Yan et al. 2012	AAGAGCAGACTGATGCATGG<br>ATATTGGAGCAGAACCACCC
Nitrite reductase<br>*ZmNIR*	N metabolism	GRMZM2G079381	AGGTGGCGGACATCGGCTTC<br>ACGGCACGGACTTCCTGTAGAC
Nitrate reductase 1<br>*ZmNR1*	N metabolism	GRMZM2G076723	TGCTTCTGGTCCGTCGAGGTGG<br>ACACGTTCACCTTCACCTTGAA
Nitrate reductase 2<br>*ZmNR2*	N metabolism	Bowsher et al. 1991	ACTGGTGCTGGTGCTTCTGGTCC<br>ATGCCGATCTCGCCCTTGTGC
High affinity nitrate transporter<br>*ZmNAR2.2*	N metabolism	GRMZM2G163494	GCTGGAGGTGACCCTCTGCTACG<br>TGCCGGGCGATCCTGAACTGG
Phosphate transporter 1;1<br>*ZmPht1*	P uptake	GRMZM2G326707	CGTAGTACGTGTGTGATAGTCTGG<br>TATTATCACACGTGGACCTCTACC
Phosphate transporter 1;3<br>*ZmPht3*	P uptake	GRMZM2G112377	GCCTTCCGTTACGTCATTGT<br>AGCACGTCTCTGATCCCATC
Phosphate transporter 1;4<br>*ZmPht4*	P uptake	GRMZM2G170208	ACCGGCTACCCTCACCTACT<br>CTACCTTCTTGGCGTCCTTG
Phosphate transporter 1;8<br>*ZmPht8*	P uptake	GRMZM2G045473	CCTGGAGGAGATGTTCAGGA<br>AAGACGGTGAACCAGTAGCC
Phosphate transporter 1;9<br>*ZmPht9*	P uptake	Xu et al. 2021	CATTGTCACGCTCGTCATCT<br>GGTGGAGTTGAAGTGGTCGT
Glutathione Reductase<br>*ZmGSR1*	Stress-related	Xu et al. 2022	CCAATAGGGTCAACCTGACACCAG<br>TCCATACTCTTCAATTGCCTGCTC
Glutamin Synthetase<br>*ZmGS2*	N metabolism	Li et al. 2021	TATAAACCGGTCCGCGACA<br>CGATGAATCAAAGACAGCCGT
β-1,3-glucanase<br>*ZmPR1*	Stress-related	Cao et al. 2023	AACCTTCTTGGCACCACCCT<br>GTTGGTGTCGTGGTCGTAGT
Endochitinase PR4<br>*ZmPR4*	Stress-related	NM_001157282.1	TGATGGATAGATGGCGATTGC<br>AGAATTGACACCGCCAAACC
Lipoxygenase 1<br>*ZmLOX1*	Stress-related	Ogunola et al. 2015	CACTCGAGCTCGTCAAGGAT<br>TCCAACCTGTCTTGTCCTCTTT
Superoxide dismutase 2<br>*ZmSOD2*	Stress-related	Gautam et al. 2023	CACCAACGGCTGCATGTC<br>ATGCTCCTTGCCAACAGGAT
Superoxide dismutase 4<br>*ZmSOD4*	Stress-related	XM_008653632.4	CACCAACGGCTGCATGTC<br>ATGCTCCTTGCCAACAGGAT
1-Aminocyclopropane-1-carboxylate synthase<br>*ZmACS6*	Stress-related	NM_001143622.2	GTGCTCATCACCAACCCTTC<br>ACGAAGTCCACCAGCATCTC
Defensin-like protein 1<br>*ZmDef1*	Stress-related	NM_001329491.1	TGCTGCTCCTCATCGTCGTTGC<br>TTGCCGCCGCCGTAGCCTTC

Defensin-like protein 2 *ZmDef2*	Stress-related	NM_001153491.2	AGTCCAGGGCGACCGTGTG<br>CGAGTGGTGCTGGCTCTTGC
WRKY transcription factor *ZmWRKY106*	Stress-related	GRMZM2G013391	GCTCGTCACCTACACCTTCG<br>AGCTTTCGTCCTCCTCTGC
WRKY transcription factor *ZmWRKY17*	Stress-related	GRMZM2G102583	TTTTTCTTCTTCCGCTGTTCTACTC<br>TCAGATCGAGGGTCGTCATCT
WRKY transcription factor *ZmWRKY33*	Stress-related	GRMZM2G148087	GTGGTCCAGACGATGAGCGACAT<br>GCTGCTCAGCATCTCCAGGGTGT
WRKY transcription factor *ZmWRKY40*	Stress-related	Hu et al. 2021	CTACTTCCGCTGCTCCTTCG<br>TGCTGCTGCTGGTGCTGCT
WRKY transcription factor *ZmWRKY58*	Stress-related	GRMZM2G147880	AGGAAGTGGAGGAGGCGAACA<br>GGATGGCTTGCGCTTGC
Ethylene-responsive factor-like protein *ZmERF1*	Stress-related	NM_001111800.2	ACTTCCCCAGCGACACCTC<br>TGACCTCGTCGGACACCTGA
Ethylene-responsive transcription factor *ZmEREB58*	Stress-related	NM_001176924.1	GACGGCGACAAGAAGCGA<br>CGGTGCCAGGACGACG
MYB transcription factor *ZmMYB30*	Stress-related	GRMZM2G087955	CTCCTTGTCGTTGTCCCTCT<br>CTTGCTTGTGCTTGAGGTGT
MYB transcription factor *ZmMYB36*	Stress-related	GRMZM2G139284	GGTGTTCGAGTACGAGACGA<br>ACAGGACGGTGGAAGTGG
MYB transcription factor *ZmMYB95*	Stress-related	GRMZM2G139284	CTCGTCTTCTCTCCGCTACC<br>TAGTCGACGACAACGAGTGG
